# The American Journal of Urology

**Published:** 1904-11

**Authors:** 


### The American Journal of Urology.

THE *American Journal of Urology* has made its appearance, and the genitourinary specialists at last have a journal devoted exclusively to their interests without a dermatologic attachment. The first number, October, 1904, is a most excellent issue of a new journal from every viewpoint. It is typographically a handsome journal, and contains much valuable material concerning the specialty it represents. It is a credit to its publishers, the Grafton Press, and its editor, Dr. Henry G. Spooner, of New York.

The leading features of the initial number are articles by Kelly, of Baltimore; Ayres, of New York; and Cathelin, of Paris, all eminent in the field of genitourinary diseases. In addition there is a most excellent and intelligently arranged abstract department, which will be a permanent feature and which will be of inestimable value to the genitourinary specialist in keeping track of the literature of the world. The journal has been made the official organ of the American Urological Association and several other genitourinary societies, and this in itself makes it a most valuable addition to medical literature.

The editorial board consists of the following well-known specialists: Arthur Bevan, M.D., of Chicago; Follen Cabot, M.D., of New York; A. E. Gallant, M.D., of New York; Orville Horwitz, M.D., of Philadelphia; Howard A. Kelly, M.D., of Baltimore; Gustav Kolischer, M.D., of Chicago; Robert T. Morris, M.D., of New York; William H. Porter, M.D., of New York; Robert Bruce Preble, M.D., of Chicago, and Hugh H. Young, M.D., of Baltimore.

